# Reference gene considerations for toxicological assessment of the flame retardant triphenyl phosphate in an in vitro fish embryonic model

**DOI:** 10.1002/jat.4698

**Published:** 2024-09-18

**Authors:** Logan Germain, Delaine Pereira, Louise M. Winn

**Affiliations:** ^1^ Department of Biomedical and Molecular Sciences Queen's University Kingston Canada; ^2^ School of Environmental Studies Queen's University Kingston Canada

**Keywords:** embryonic development, endocrine disruption, flame retardant, reference gene, RT‐qPCR, Triphenyl phosphate

## Abstract

The reliability of relative quantification RT‐qPCR depends upon the gene of interest being normalized to one or more reference genes, with the assumption that the chosen reference genes do not experience altered expression with experimental conditions. The correct choice of stable reference genes is critical when investigating alterations to gene transcript levels following exposure to endocrine and metabolic disrupting chemicals, such as the flame retardant triphenyl phosphate (TPhP). This study assessed the stability of eight reference genes following TPhP exposure in embryonic cells derived from rainbow trout ( *Oncorhynchus mykiss* ). The genes β‐actin (*actb*) and 18s rRNA (*18s*) were stable, while glyceraldehyde‐3‐phosphate dehydrogenase (*gapdh)* relative expression was found to be increased. *gapdh* is a popular reference gene and has been previously used in the literature for investigating TPhP exposure in teleost fish models. We discuss the implications of *gapdh* upregulation in the context of TPhP as a metabolic disrupting chemical. Furthermore, we quantified the expression of the tumor suppressor gene *p53* following TPhP exposure in relation to different reference genes to use as an example to report on how discrepancies in findings might arise depending on the stability of the chosen reference gene.

## Abstract 2

The stability of eight reference genes following triphenyl phosphate (TPhP) exposure in embryonic cells derived from rainbow trout ( *Oncorhynchus mykiss* ) was assessed. β‐actin (*actb*) and 18s rRNA (*18s*) were stable, while glyceraldehyde‐3‐phosphate dehydrogenase (*gapdh)* relative expression was found to be increased. *gapdh* is a popular reference gene and has been previously used in the literature for investigating TPhP exposure in teleost fish models. We discuss the implications of *gapdh* upregulation in the context of TPhP as a metabolic disrupting chemical.

## 1 INTRODUCTION

Teleost fish such as rainbow trout (*Onchorhynchus mykiss*) and zebrafish (*Danio rerio*) are increasingly being used as model organisms in the fields of ecotoxicology, human disease and reproduction (Best et al., 2018; Schartl, 2014; Thorgaard et al., 2002). Teleost fish exhibit remarkably high sensitivity to changes in their environment, which makes them important species in basic and translational research to explore the effects of environmental toxicants (Schartl, 2014). Furthermore, the use of embryos derived from teleost fish such as rainbow trout can offer alternative models for studying the impacts of the environmental toxicant exposure on normal embryonic development. The time and resources required to conduct classic in vivo toxicity tests is a barrier for assessing every emerging chemical of concern in a timely fashion. Therefore, the use of alternative in vitro models can contribute to the Adverse Outcome Pathway (AOP) framework by being a useful tool to gain basic mechanistic insights of molecular events that lead to altered biological outcomes due to toxicant exposures (Ankley et al., 2010).

One such toxicant is the flame retardant and plasticizer triphenyl phosphate (TPhP). TPhP is not chemically adhered to products and has a tendency to leach into its surroundings (Carlsson et al., 2000). Flame retardants are often present in both indoor and outdoor environments and can be long‐range transported via water systems and atmospheric processes to widespread remote ecosystems (Fu et al., 2021; Möller et al., 2012). TPhP is highly pervasive in water systems (Sundkvist et al., 2010), which poses a concern for aquatic wildlife exposures. TPhP is widely accepted to act as an endocrine disrupting chemical (EDC) in estrogenic, thyroid, and metabolic pathways (Ji et al., 2020, 2022; Kim et al., 2015; Philbrook et al., 2018; Wang et al., 2019), with capabilities of altering epigenetic modifications in in vitro and in vivo models (Germain & Winn, 2024; Shafique et al., 2023). There remains much conflicting evidence in the literature regarding the specific EDC effects of TPhP. For example, studies conducted in a variety of in vitro and in vivo models have found TPhP to have pro‐estrogenic effects (Ji et al., 2020, 2022; Kojima et al., 2013; Zhang et al., 2015), while others have demonstrated anti‐estrogenic effects (Ji et al., 2020; Ma et al., 2021). Estrogenic and metabolic signaling during embryonic development is tightly regulated, and one important regulator of these processes is the tumor suppressor gene *p53* (Kung & Murphy, 2016; Sengupta & Wasylyk, 2004).

Reverse transcription quantitative polymerase‐chain reaction (RT‐qPCR) is a heavily used technique in the field of toxicology to measure changes to gene transcript levels in response to exposure to environmental EDCs. The reliability of relative quantification RT‐qPCR depends upon the gene of interest being normalized to one or more reference gene(s), with the assumption that the chosen reference genes do not have altered expression with experimental conditions (Liu et al., 2018; McCurley & Callard, 2008; Shekh et al., 2017). Common reference genes used in toxicological studies include *actb*, *gapdh* and *18s*, though these genes have been shown to be altered under certain experimental conditions (Radonić et al., 2004). The choice of reference genes can have profound implications on the findings of a study. For example, McCurley and Callard (2008) demonstrated that the relative expression of the aromatase enzyme (*cyp19a1b)* following exposure to an estrogen receptor antagonist in zebrafish showed dramatically different results depending on the reference gene used for normalization, including up‐ and down‐regulation from the same samples. The reporting of incorrect findings due to improper normalization strategies can be mitigated by performing reference gene selection panels prior to gene of interest investigations, as well as normalizing to more than one reference gene.

The correct choice of stable reference genes will be critical for future studies investigating alterations to gene transcript levels in endocrine and metabolic pathways following TPhP exposure. This study aimed to assess and compare the stability of a panel of eight different commonly used reference genes following TPhP exposure in embryonic cells derived from rainbow trout. We conclude that *actb* and *18s* are the two most appropriate reference genes, while *gapdh* demonstrated the lowest stability and in fact, *gapdh* expression was found to be increased. We discuss the implications of *gapdh* upregulation in the context of TPhP as a metabolic disrupting chemical. Furthermore, we quantified the expression of the tumor suppressor gene *p53* in relation to different reference genes and use this as an example to report on how discrepancies in findings might arise depending on the stability of the chosen reference gene.

## 2 MATERIALS AND METHODS

### 2.1 Cell culture and exposure model

This study used the immortalized cell line STE‐137, derived from pooled steelhead trout (*Onchorhynchus mykiss irideus*) embryonic tissue. This cell line was privately provided to us by the United States Geological Survey Western Fisheries Research Centre (Seattle, WA, USA) but can be acquired from the European Collection of Authenticated Cell Cultures (95122020). This cell line was first characterized by Lannan et al. (1984) and more recently reviewed in‐depth by Bols et al. (2017). STE‐137 cells were grown in a supplemented growth media containing 88% Leibovitz's L‐15 media (Sigma‐Aldrich, St. Louis, MO, USA), 10% fetal bovine serum from Gibco (Grand Island, NY, USA), 1% L‐glutamine (Sigma‐Aldrich,), and 1% penicillin–streptomycin solution (Wisent Bioproducts, Saint‐Jean‐Baptiste, QC, Canada). The cells were grown in T25 culture flasks with vented caps at 18°C. Experiments were carried out when cell populations were at 80–90% confluency which was approximately 250,000 cells/ml at passage numbers 20–30. Six biological replicates were analyzed in technical triplicate for each gene expression comparison. A biological replicate was defined as a distinct population of cells within a culture flask that were each exposed to TPhP on a different day within an exposure group. Each population of cells originated from the same batch.

### 2.2 Preparation of test chemical solution

Triphenyl phosphate (TPhP) (CAS No. 115‐86‐6) was purchased from Sigma‐Aldrich (St. Louis, MO, USA). A working stock solution of 100 mM TPhP was dissolved in dimethyl sulfoxide (DMSO) from Sigma‐Aldrich (St. Louis, MO, USA) and stored in the dark at room temperature. This working stock solution was diluted into the supplemented growth media to achieve the desired concentrations. The final concentration of DMSO in the supplemented growth media of all exposure groups in this study, including in the vehicle control group, was 0.08% (v/v). Following a 24 h initial attachment period, 5 ml of fresh media was added to T25 flasks and cell populations were exposed to sub‐lethal concentrations of TPhP (0, 40, or 80 μM for 24 h) as previously established by Germain and Winn (2024). This exposure regimen has previously been shown to alter the epigenome in this in vitro model (Germain & Winn, 2024).

### 2.3 RNA isolation and cDNA synthesis

Following the 24 h exposure period, cells were harvested, and RNA was extracted using the Aurum™ Total RNA Kit (Bio‐Rad, Hercules, CA, USA) following manufacturer's instructions. This kit contains a DNase I enzyme. RNA purity was assessed using the NanoDrop 2000 Spectrophotometer (Thermo Fischer Scientific, Waltham, MA, USA), assessed for an appropriate 260/280 nm absorbance ratio (>2) to indicate purity and the concentration was recorded. A total of 50 ng/μl of RNA per reaction was then reverse transcribed into cDNA using the iScript™ cDNA Synthesis Kit (Bio‐Rad, Hercules, CA, USA) as per the manufacturer's instructions. cDNA was stored at −80°C and diluted to 2 ng/μl prior to use in subsequent steps.

### 2.4 Primer design

The mRNA transcript sequences for each gene of interest were acquired from the NCBI Gene database. Primers for each transcript, as described in Table 1, were designed using the free, online PrimerQuest™ Tool by Integrated DNA Technologies. Primer sequences were assessed for specificity both in silico using NCBI‐BLAST online software, and with qualitative melting curve analysis, which can be found in the Supplementary Materials. Primer pairs were assessed for efficiencies between 90 and 110% using Bio‐Rad CFX Maestro Software (Bio‐Rad, Hercules, CA, USA).

**TABLE 1:** Gene and primer information.

Gene name	Gene symbol	Primer sequences (5′‐3′)	Accession #	Tm (°C)	Primer efficiency (%)	R^2^
TATA‐binding protein	*tbp*	*Fwd*: CCA TTC GGT TAG AGG GAC TTG	XM_021581318.2	*Fwd*: 55.1	106.7	0.997
		*Rev*: CAA CAG GAC AAT TCT GGG TTT G		*Rev*: 54.4		
β‐2‐microglobulin	*b2m*	*Fwd*: AGG ATC TGG AGC AGG ACA TA	L49056	*Fwd*: 54.8	108.7	0.981
		*Rev*: CAC CTT GGC ACA AAG TGT TAT C		*Rev*: 54.4		
18S ribosomal RNA	*18*s	*Fwd*: CTG AGA AAC GGC TAC CAC ATC	XR_005038417.1	*Fwd*: 55.5	105.6	0.999
		*Rev*: GCC TCG AAA GAG TCC TGT ATT G		*Rev*: 55.3		
Elongation factor 1‐alpha	*ef1a*	*Fwd*: CGG AGG CAT TGA CAA GAG AA	NM_001124339.1	*Fwd*: 55.0	108.7	0.995
		*Rev*: CAG GGA AAT GTC GAT GGT GAT A		*Rev*: 54.5		
Tubulin, β‐2b	*tubb2b*	*Fwd*: GCT CTC TAC GAC ATC TGC TTC	XM_021605618.2	*Fwd*: 55.0	109.4	0.995
		*Rev*: GTT GTG ACA CCG CTC ATA GT		*Rev*: 54.9		
Glucose‐6‐phosphate dehydrogenase	*g6pd*	*Fwd*: GAA CAG GGT GAT TGT GGA GAA G	XM_021615620.2	*Fwd*: 55.6	104.2	0.989
		*Rev*: GGT AGT GGT CTA TGC GGT AGA T		*Rev*: 55.6		
Glyceraldehyde‐3‐phosphate dehydrogenase	*gapdh*	*Fwd*: GAA GTA CGA GAA CTC CCT CAA G	NM_001124209.1	*Fwd*: 54.5	109.5	0.993
		*Rev*: GTG CTC ATC AGA CCC TCA AT		*Rev*: 54.6		
β‐actin	*actb*	*Fwd*: ACC CAC ACA GTA CCC ATC TA	NM_001124235.1	*Fwd*: 55.0	93.6	0.998
		*Rev*: TCA GGG TCT TCA TCA GGT AGT		*Rev*: 54.8		
Tumor protein p53	*p53*	*Fwd*: TCA GAA ATG CCT CAC CAA GAG	NM_001124692.1	*Fwd*: 54.8	109.6	0.996
		*Rev*: TCT CCT CCT TCA CCA GTA GTT		*Rev*: 54.7		

### 2.5 Real‐time quantitative PCR

All RT‐qPCR analysis was done using the Bio‐Rad CFX Maestro software (Version: 5.3). The software outputs mean relative expression of each exposure group compared to control with an associated standard error of the mean. RT‐qPCR was performed using the iTaq™ Universal SYBR Green Supermix (Bio‐Rad, Hercules, CA, USA) in a total reaction volume of 10 μl containing 2.5 μl of cDNA at 2 ng/μl for relative gene expression analysis or at 1 ng/μl for reference gene panel in 96‐well plates. The amplification program consists of an initial denaturation step at 95° C for 30 s, then 39 cycles of denaturation at 95° C for 5 s, annealing/extension at 60° C for 30 s, with a final melt curve analysis (65–95° C, increasing by 0.5° C every 5 s). Wells containing no cDNA were included in each RT‐qPCR run as negative controls to ensure no contamination was present. The baseline subtraction level was auto calculated for each RT‐qPCR reaction by the Bio‐Rad CFX Maestro software. A standard deviation of ≤0.5 cycles between Cq values of technical replicates was permitted.

### 2.6 Statistical analysis

Reference gene stability across all experimental conditions was assessed using the Reference Gene Selector Tool in Bio‐Rad CFX Maestro software. This tool is based upon the geNorm algorithm and the reference gene stability protocol described by Vandesompele et al. (2002). Reference gene stabilities were assessed using the same pooled sample composed of six biological replicates, measured in technical triplicate for each concentration point and gene. In brief, the geNorm algorithm evaluates pairwise variations between each reference gene and all other genes to generate an average expression stability measure (M) for each gene (Vandesompele et al., 2002). The relative expression of *p53*, *gapdh*, *actb* and *18s* genes were quantified by the Bio‐Rad CFX Maestro software version 2.3 using the ∆∆ Cq method with a correction for primer efficiency. Each relative gene expression analysis had six distinct biological replicates at each concentration point, measured in technical triplicate. The reference gene that was used for normalization depended on the comparison being made and can be found in the figure caption. Comparisons of the relative gene expression in TPhP exposure groups was performed using ordinary one‐way ANOVAs followed by Dunnett's multiple comparisons test or an un‐paired t‐test on GraphPad Prism 9.0 software (GraphPad, San Diego, CA, USA). Statistical significance is defined as a *p*‐value <0.05.

## 3 RESULTS

### 3.1 Expression stability of reference genes

**FIGURE 1:**
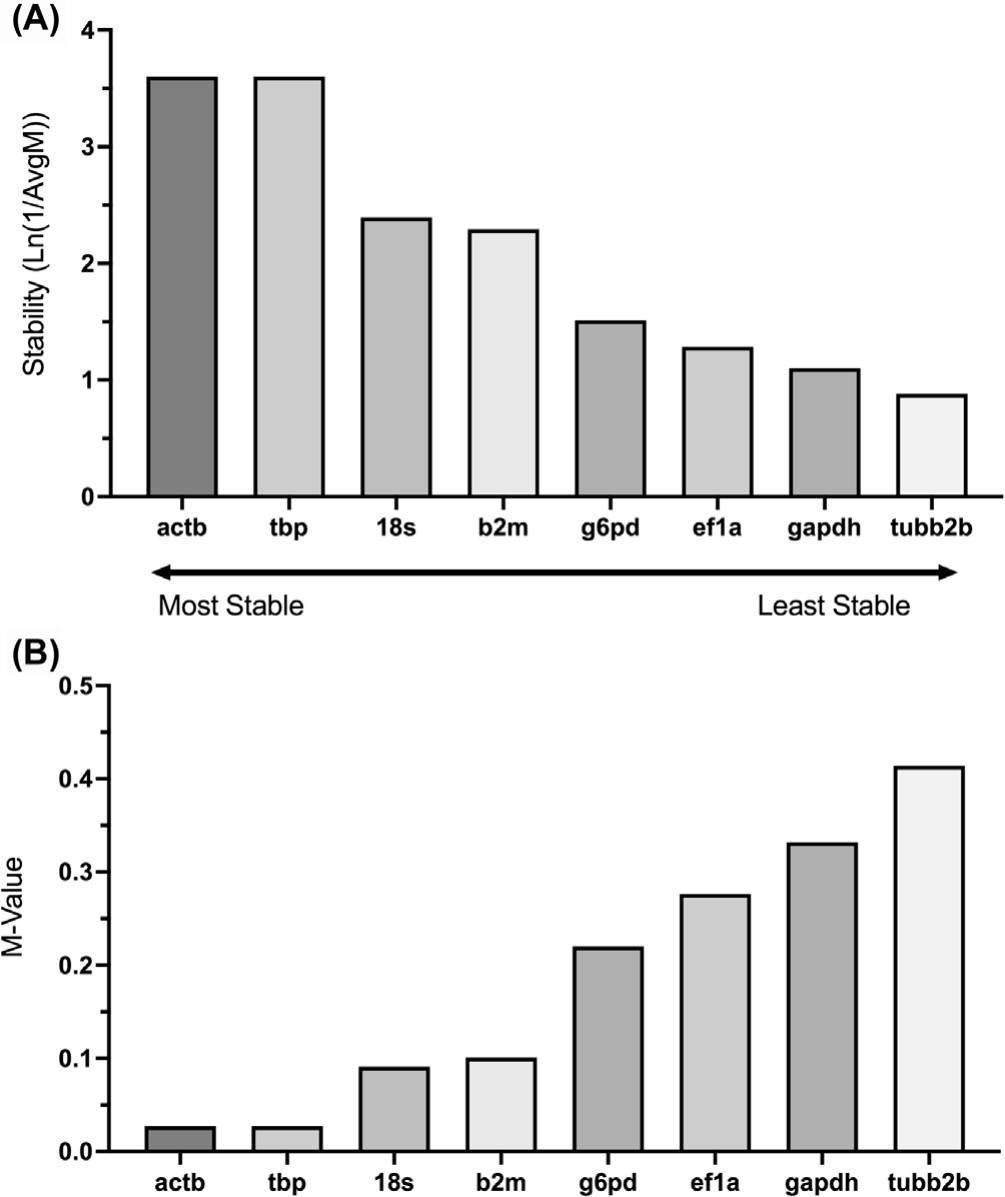
Reference gene rankings according to their average reference stability values across 0, 40, and 80 μM of TPhP exposure in STE‐137 embryonic cells. A) Average gene expression stability and B) corresponding M‐value of each tested reference gene, as determined with RT‐qPCR analysis using the Bio‐Rad Reference Gene Selector Tool based on the geNorm algorithm. The M‐value of a gene is inversely proportional to its stability. Each gene was assessed from the same pooled sample of n = 6 biological replicates for each TPhP concentration point and measured in technical triplicate on a 96‐well plate.

Reference gene stability rankings were generated on the Bio‐Rad CFX Maestro software, based on the geNorm algorithm, and can be seen on Figure 1. The genes *actb, tbp*, and *18s* demonstrated the highest stabilities (3.6, 3.6 and 2.4, respectively) and lowest corresponding M‐value (0.03, 0.03 and 0.09, respectively). Conversely, *gapdh* and *tubb2b* demonstrated the lowest stabilities (1.1 and 0.88, respectively) and highest corresponding M‐value (0.33 and 0.41). The M‐value of a gene is inversely proportional to its stability.

### 3.2 Mean expression and standard error of reference genes

**FIGURE 2:**
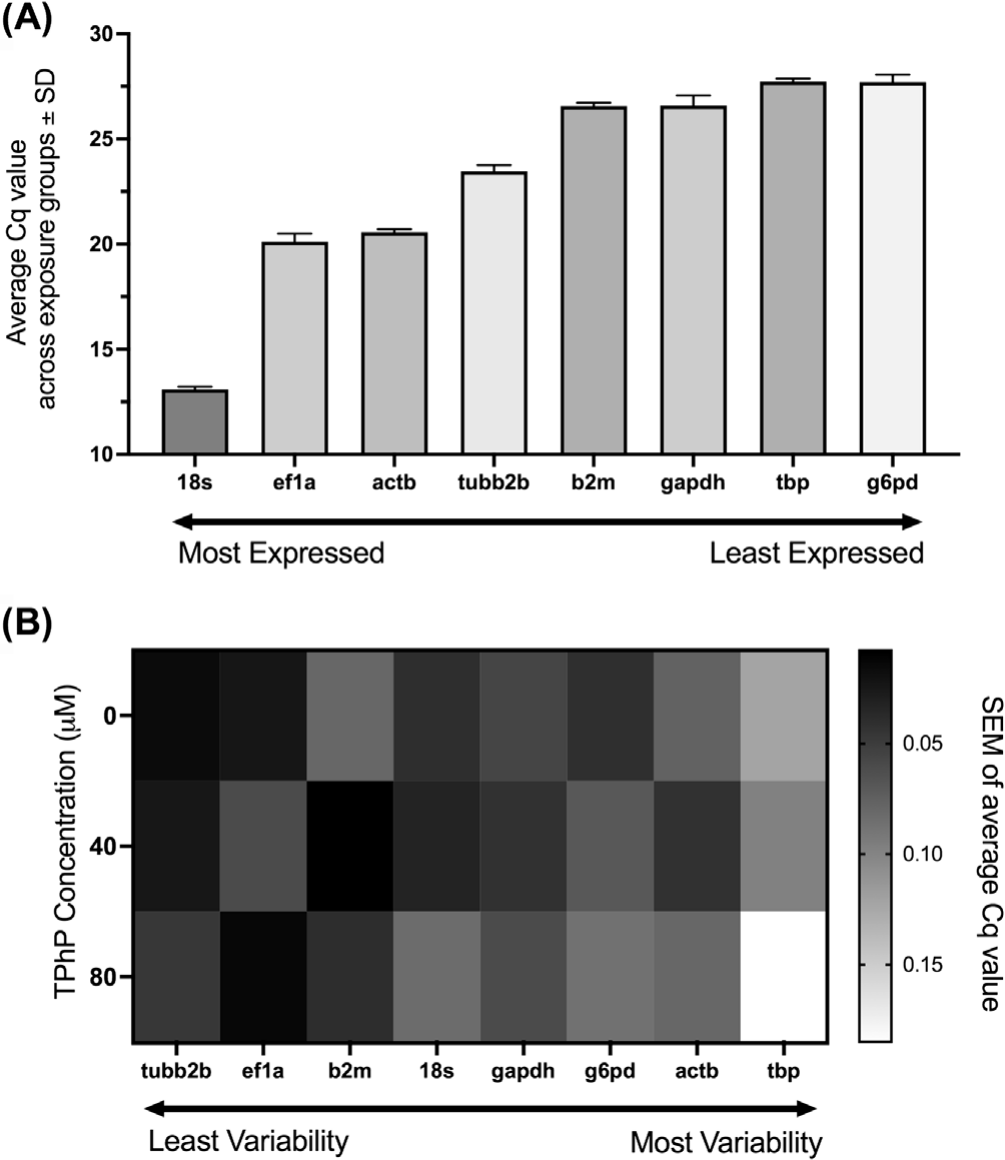
Expression and variability of each tested reference gene in STE‐137 embryonic cells exposed to 0, 40, and 80 μM of TPhP. Each gene was assessed from the same pooled sample of n = 6 biological replicates at each TPhP concentration point and measured in technical triplicate on a 96‐well plate. A) Average Cq value of each tested reference gene across all three exposure groups. A lower Cq value indicates more gene transcript present. B) The standard error of the mean between technical replicates for each tested reference gene at 0, 40, or 80 μM of TPhP.

The reference genes *18s* and *ef1a* demonstrated the highest mean expression across all three exposure groups, according to their Cq values of 13.1 and 20.1 (Figure 2A and Table 2), where a lower Cq value indicates more gene transcript present in a sample. The genes *tbp* and *g6pd* had the lowest mean expression, based on their mean Cq values of 27.72 and 27.69 (Figure 2A and Table 2). Each gene was assessed from the same pooled sample of six biological replicates for each TPhP concentration point and measured in biological triplicate on one 96‐well plate. The standard error of the mean between technical replicates was the lowest in *tubb2b* and *ef1a* genes, and the highest being in *tbp* which was twice as high as the second, *actb* (Figure 2B).

**TABLE 2:** Reference gene panel Cq information.

Gene symbol	TPhP exposure concentration (μM)	Mean Cq of each exposure group	Mean Cq across exposure groups	Cq SEM	Mean Cq SEM across exposure groups
*18s*	0	13.32	13.09	0.03951	0.051228
*18s*	40	12.90		0.03154	
*18s*	80	13.06		0.08263	
*b2m*	0	26.86	26.56	0.07878	0.041818
*b2m*	40	26.32		0.00773	
*b2m*	80	26.52		0.03895	
*actb*	0	20.73	20.56	0.07557	0.065299
*actb*	40	20.28		0.04160	
*actb*	80	20.68		0.07873	
*ef1a*	0	20.87	20.11	0.02263	0.031669
*ef1a*	40	19.63		0.05961	
*ef1a*	80	19.85		0.01276	
*g6pd*	0	28.38	27.69	0.03996	0.064802
*g6pd*	40	27.19		0.06862	
*g6pd*	80	27.54		0.08583	
*gapdh*	0	27.49	26.58	0.05507	0.051865
*gapdh*	40	25.87		0.04109	
*gapdh*	80	26.40		0.05944	
*tbp*	0	27.92	27.72	0.12147	0.134342
*tbp*	40	27.42		0.09675	
*tbp*	80	27.82		0.18480	
*tubb2b*	0	23.29	23.46	0.01522	0.027936
*tubb2b*	40	23.04		0.02318	
*tubb2b*	80	24.04		0.04541	

Gene, exposure group and Cq information for all eight tested reference genes in STE‐137 embryonic cells. Cq values were measured with a pooled sample containing n = 6 biological replicates, measured in technical triplicate.

### 3.3 Relative expression of *gapdh, actb, 18s*, and *p53*

**FIGURE 3:**
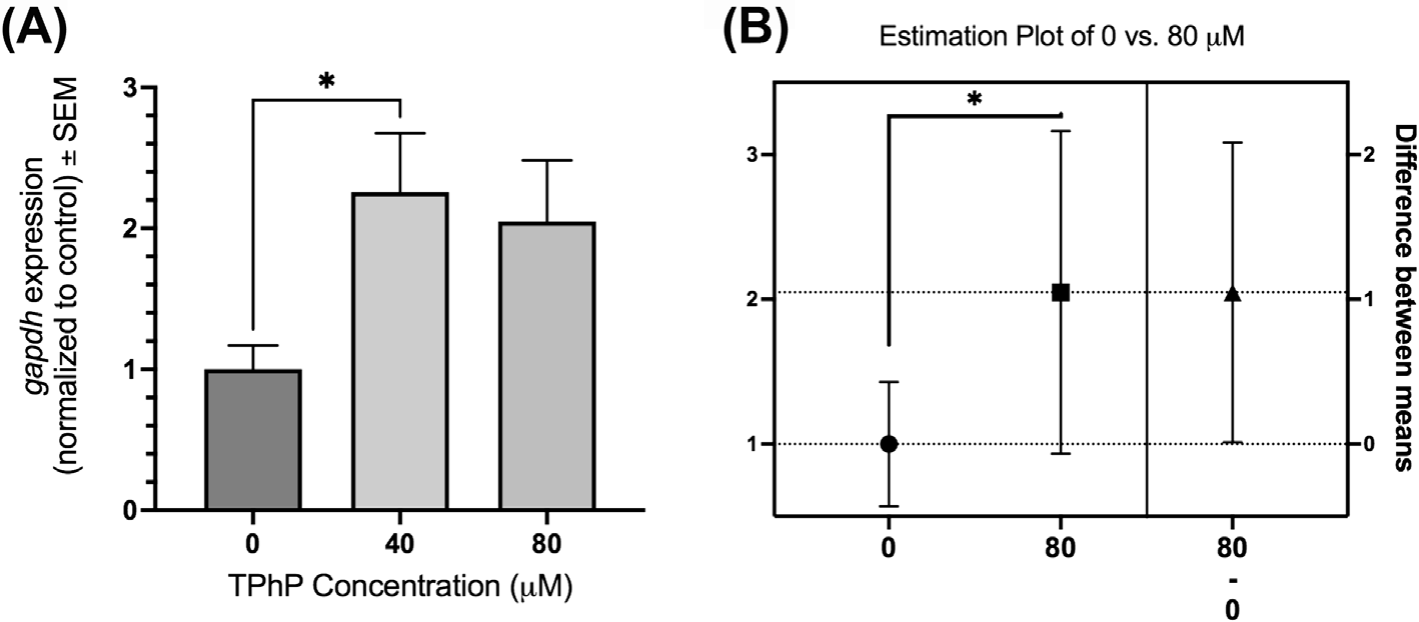
Relative gene expression of *gapdh* in STE‐137 embryonic cells exposed to 0, 40, and 80 μM of TPhP. Assessed using RT‐qPCR analysis with n = 6 biological replicates measured in technical triplicate and normalized to the reference genes *actb* and *18s*. A) *gapdh* expression is significantly increased at 40 μM of TPhP using a one‐way ANOVA and Dunnett's multiple comparisons test relative to control (*p* = 0.0476). B) *gapdh* expression is significantly increased at 80 μM of TPhP using an unpaired t‐test relative to control (*p* = 0.0478).

The expression of *gapdh* was assessed at 40 and 80 μM of TPhP relative to control and normalized to both *actb* and *18s* genes. Gene expression of both exposure groups relative to control was assessed using a one‐way ANOVA with Dunnett's multiple comparisons test post‐hoc. We found that *gapdh* at 40 μM of TPhP shows a significant increase of mean expression compared to control (225%), while at 80 μM shows a non‐significant increase of mean expression compared to control (200%) (*p* > 0.05) (Figure 3A). Though, when directly comparing the 80 μM exposure group to the control group with an unpaired t‐test, the difference in means is statistically significant (*p* < 0.05) (Figure 3B). Therefore, *gapdh* expression is increased following TPhP exposure, though at which concentration depends on the specific statistical test being conducted.

**FIGURE 4:**
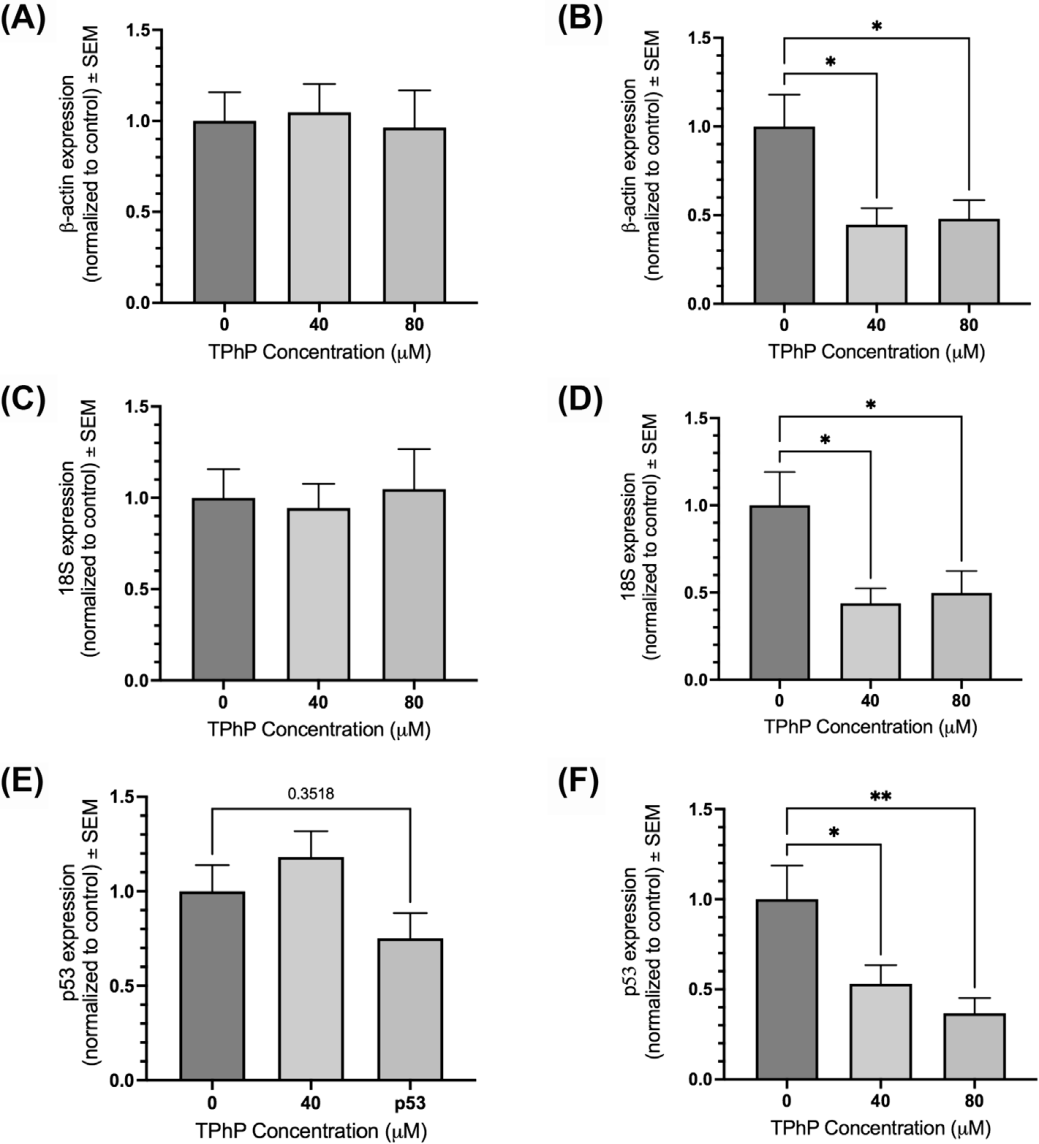
Relative gene expression of the reference genes *actb* and *18s*, and the tumor suppressor gene p53 exposed to 0, 40 or 80 μM of TPhP, when normalized to different reference genes. Assessed using RT‐qPCR analysis with n = 6 biological replicates measured in technical triplicate. A) *actb* expression remains unchanged relative to control when normalized to *18s*. B) *actb* expression is significantly decreased at 40 and 80 μM TPhP relative to control when normalized to *gapdh* (*p* = 0.018 and 0.025, respectively). C) *18s* expression remains unchanged relative to control when normalized to *actb*. D) *18s* expression is significantly decreased at 40 and 80 μM TPhP relative to control when normalized to *gapdh* (*p* = 0.0237 and 0.0421, respectively). E) *p53* expression shows no significant differences relative to control when normalized to both *18s* and *actb* (*p = 0.3518*). F) *p53* expression is significantly decreased at 40 and 80 μM TPhP relative to control when normalized to *gapdh* (*p* = 0.044 and 0.0077, respectively).

The expression of *actb* was assessed at 40 and 80 μM of TPhP relative to control and normalized to either *18s* or *gapdh* genes. When normalized to *18s*, *actb* shows no significant changes at any concentration point compared to control (Figure 4A). When normalized to *gapdh*, *actb* shows a significant decrease in mean expression at both 40 and 80 μM of TPhP (45% and 48% compared to control) (Figure 4B).

The expression of *18s* was assessed at 40 and 80 μM of TPhP relative to control and normalized to either *actb* or *gapdh* genes. When normalized to *actb*, *18s* shows no significant changes at any concentration point compared to control (Figure 4C). When normalized to *gapdh*, *18s* shows a significant decrease in mean expression at both 40 and 80 μM of TPhP (44% and 50% compared to control) (Figure 4D).

The expression of *p53* was assessed at 40 and 80 μM of TPhP relative to control and normalized to either *18s* and *actb*, or to *gapdh* alone. When normalized to *actb/18s*, *p53* shows no significant changes at any concentration point compared to control (Figure 4E). When normalized to *gapdh*, *p53* shows a significant decrease in mean expression at both 40 and 80 μM of TPhP (53% and 36% compared to control) (Figure 4F).

## 4 DISCUSSION

### 4.1 Reference gene selection panel

The choice of a stable reference gene, one whose expression does not vary with experimental conditions, is a crucial step for valid interpretation of results. There does not exist one universally valid reference gene, so determination of appropriate reference genes must occur for every experimental design. No reports yet exist for reference gene selection for TPhP exposure in trout during embryonic development. This study demonstrates that gene expression of the cytoskeleton protein β‐actin (*actb*), the TATA‐box binding protein (*tbp*) and 18s rRNA (*18s*) subunit were the most stable across TPhP exposures in embryonic cells derived from trout. Conversely, the commonly used reference genes GAPDH (*gapdh)* and β‐tubulin (*tubb2b*) demonstrated the lowest stabilities, Interestingly, all eight reference genes tested here showed low enough M‐values to indicate stability (Hellemans et al., 2007; Köhsler et al., 2020; Vandesompele et al., 2002). Therefore, we recommend conducting additional comparisons between reference genes prior to use, as *actb* and *18s* expression were stable in relation to one another while *gapdh* expression was not.

The more copies of a gene transcript that exist leads to fewer cycles of amplification needed in order to reach the cycle threshold (Cq) value (Ruiz‐Villalba et al., 2021). The *18s* gene had a lower Cq value (mean of 13.1) across all exposure groups than any other gene. The *actb* gene had a mean Cq of 20.6, the third lowest of the genes tested. Conversely, the genes *tbp* and *g6pd* both showed higher Cq values (mean of 27.7), indicating lower transcript numbers.

We assessed the standard error of the mean (SEM) at 0, 40 and 80 μM of TPhP for each reference gene. Given that each gene tested at the same concentration was from the same pooled sample, variability as measured by SEM between technical replicates gives an indication of the reproducibility of that reference gene's expression. The *tbp* gene showed the largest variability, leading to its disqualification as a top reference gene selection, despite its high stability via the geNorm algorithm.

Finally, to provide additional evidence for the stability of the *actb* and *18s* genes, we analyzed the relative expression of both genes in relation to one another. We showed that *actb* expression remains unchanged across 40 and 80 μM of TPhP relative to control when normalized to *18s*. Expression of *18s* also remains unchanged across 40 and 80 μM of TPhP when normalized to *actb*. We concluded that *actb* and *18s* expression remains stable across all TPhP exposures. Using *18s* as a reference gene is extremely common in qPCR applications, though there have been reported several pitfalls of this reference gene. Typically, the expression of *18s* is higher than any gene of interest which can lead to artificially stable expression (Paolacci et al., 2009). Additionally, transcription and degradation pathways differ between rRNA and mRNA (Paolacci et al., 2009). Therefore, we chose to use *18s* in addition to a second reference gene *actb* to mitigate any potential inaccuracies of using either gene alone.

### 4.2 Relative gene expression of *gapdh*

Though *gapdh* showed an acceptable stability according to the geNorm algorithm, and an M‐value below the acceptable cut‐off of 0.5 as described by Hellemans et al. (2007), we further investigated its relative expression. An analysis of *gapdh* gene expression normalized to *actb* and *18s* revealed that its expression was significantly increased at 40 and 80 μM of TPhP relative to control. This is a particularly interesting finding, as TPhP is known to act as a metabolic disrupting chemical. TPhP exposure has been shown to alter glucose uptake, impair glucose homeostasis, and contribute to insulin‐resistance (Cano‐Sancho et al., 2017; Wang et al., 2019; Yue et al., 2023). GAPDH is a key enzyme in glycolysis, converting glyceraldehyde‐3‐phosphate to 1,3‐biphosphoglycerate in the presence of NAD + and inorganic phosphate (Chaudhry & Varacallo, 2023). Beyond this function, the *gapdh* gene has been shown to be involved in regulating cell proliferation, DNA damage repair, oxidative stress responses, and overexpression is even thought to enhance cancer cell fate in a variety of in vitro and in vivo models (Nicholls et al., 2012; Tristan et al., 2011; Zhang et al., 2015). Indeed, TPhP exposure has been found to promote colorectal cancer cell line growth in vitro (Hong, Li, et al., 2022). Insulin signaling increases *gapdh* transcription through activation of the PI3K/AKT pathway (Baba et al., 2010; Zhang et al., 2015). It is possible that TPhP exposure alters insulin signaling, which upregulates *gapdh*. Further studies are required to verify this mechanism.

The increased expression of *gapdh* following TPhP exposure is of particular concern given its widespread use as a reference gene across various models including rodent, human, and teleost fish cell lines (An, Du, et al., 2023; An, Jiang, et al., 2023; Chen et al., 2024; Fan et al., 2022; Hong, Jiang, et al., 2022; Liu et al., 2013; Shi et al., 2019). For example, research conducted on zebrafish larvae by Fan et al. (2022), explored the combined effects of titanium dioxide nanoparticles and TPhP exposure on neurodevelopment. This study revealed a decrease in neuronal development marker genes which were normalized to *gapdh*, attributing these changes to TPhP‐induced neurotoxic effects. However, our findings challenge the reliability of using *gapdh* as a reference gene under TPhP exposure conditions in teleost fish models. Thus, the relative expression levels of these genes of interest may have appeared lower than their true values. The possibility that *gapdh* expression is altered following TPhP exposure raises concerns that studies which do not report validation of their reference gene stability may be drawing inaccurate conclusions.

In this study, we demonstrate that the genes *actb* and *18s* are superior to *gapdh* as reference genes used for normalization of genes of interest following TPhP exposure in trout embryonic cells. To further highlight the implications of this finding, we show that *actb* and *18s* gene expressions are stable relative to one another across TPhP exposures. However, when normalized to *gapdh*, their relative expressions are artificially significantly decreased. The choice of reference gene can drastically change the findings of a study, causing even stable genes to appear altered under the same experimental conditions.

### 4.3 Relative gene expression of *p53*

We investigated the relative gene expression of the tumor suppressor protein *p53* and used different reference genes to demonstrate the importance of proper selection. The *p53* gene is frequently found to be dysregulated in many kinds of cancers and has been shown to be modulated by EDC exposure (Lee et al., 2008). Specifically, it has been shown that estrogen exposure to zebrafish embryos resulted in decreased p53 gene expression (Santos et al., 2014), and, therefore, we speculated that p53 may be altered following TPhP exposure in our model. However, *p53* gene expression at 40 and 80 μM of TPhP was not found to be significantly different relative to control when normalized to *actb* and *18s*. This finding suggests that the *p53* gene is not a regulator at play with TPhP endocrine disruption, though species‐specific and concentration‐dependent differences may exist. Interestingly, p53 protein expression was found to be increased following TPhP exposure in mouse embryonic stem cells via Western blot analysis (Qi et al., 2019), though this expression was normalized against β‐tubulin protein expression. Gene expression of the β‐tubulin‐2b gene (*tubb2b*) was the least stable reference gene of all those tested in this study, which may account for the discrepancy in these findings. We highlight how improper reference gene selection can lead to drawing incorrect conclusions by showing that *p53* gene expression appeared significantly decreased at 40 and 80 μM of TPhP relative to control when normalized to *gapdh* but showed no significant differences when normalized *actb* and *18s*. It is conceivable that a study would use *gapdh* under the impression that it is a stable reference gene, and inaccurately report a significant decrease of *p53*.

### 4.4 Significance to the AOP framework

The AOP framework connects molecular events, such as ligand‐receptor interactions, to a biological adverse outcome such as impaired development, reproduction or disease though a chain of intermediate steps at a cellular or tissue level (Andersen & Krewski, 2010). The establishment of basic mechanistic insights to these molecular events and subsequent cellular responses is well‐suited to the use of in vitro models. The use of in vitro cell models to investigate the early steps of a given chemical exposure AOP can reduce the burden on in vivo testing and expedite the process of establishing an AOP (Ankley et al., 2010). In this study, we establish that at 80 μM of TPhP exposure, embryonic cells experience a significant increase in *gapdh* gene expression. An in vivo study has previously shown that significant changes to hepatic carbohydrate and lipid metabolism occur in zebrafish exposed to TPhP (Du et al., 2016). With additional studies being focused on other interconnecting steps between molecular initiating events, *gapdh* upregulation, disturbed carbohydrate metabolism and potential metabolic disease states in in vivo fish models, an AOP connecting TPhP exposure and the potential adverse outcome of metabolic disorders is being developed.

### 4.5 Conclusions

The findings of this study should forewarn future studies investigating the effects of TPhP exposure in fish models regarding the possibility that *gapdh* may not be a valid reference gene. We showed that *gapdh* gene expression is significantly increased following exposure to TPhP in embryonic cells derived from rainbow trout, further supporting the evidence that TPhP is a metabolic disrupting chemical. TPhP exposure has been shown to cause changes to glycolysis metabolite levels in zebrafish, increase glucose uptake in mouse adipocyte cells, increase insulin resistance, cause sex‐dependent increases in body weight, and hyperglycemia in mice (Cano‐Sancho et al., 2017; Du et al., 2016; Wang et al., 2018, 2019, Wang et al., 2020; Yue et al., 2023). In a broader context, genes involved in metabolic pathways may not be reliable as reference genes when investigating the impacts of metabolic disrupting chemicals on the transcriptome. Rather, genes involved with cellular structure or protein synthesis such as *actb* or *18s* are a better alternative in this context.

We additionally demonstrate the importance of correct reference gene selection by showing that *p53* relative expression was unchanged when normalized to the stable reference genes *actb* and *18s*, while its expression was significantly decreased when normalized to *gapdh*. Improper reference gene selection can lead to conflicting evidence in the literature and hinders progress to establish how EDCs such as TPhP impact organisms and the environment. It is important to acknowledge that species‐specific differences to EDC exposures do exist (Robaire et al., 2022), and EDCs often exhibit non‐monotonic dose responses (Vandenberg, 2014), which leads to discrepancies in findings. Nonetheless, minimizing conflicting evidence between studies with proper experimental design should be a top priority when studying toxicological compounds, especially when regulatory decisions on these compounds are based upon available literature and may be swayed towards inaction when conflicting evidence exists.

## Supporting information




**Table S1.** Primer pair melt peak images.

## Data Availability

The data that support the findings of this study are available on request from the corresponding author. The data are not publicly available due to privacy or ethical restrictions.
