# Supplementary material for: Reference gene considerations for toxicological assessment of the flame retardant triphenyl phosphate in an in vitro fish embryonic model
Source: J Appl Toxicol. 2024 Sep 18;45(2):288–97. doi: 10.1002/jat.4698 (PMC11738539; doi:10.1002/jat.4698)
Supplement: Supplementary file 1 — Table S1. Primer pair melt peak images. [file JAT-45-288-s001.docx]

**Supplementary Materials**

**Table S1.** Primer pair melt peak images.

| **Gene Symbol** | **Melt Peak** |
| --- | --- |
| *18s* | 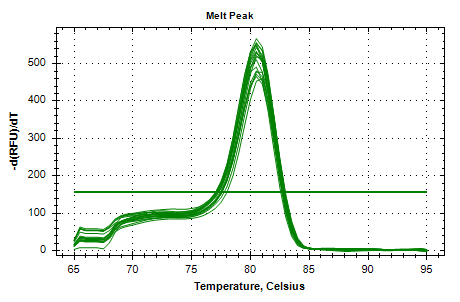 |
| *b2m* | **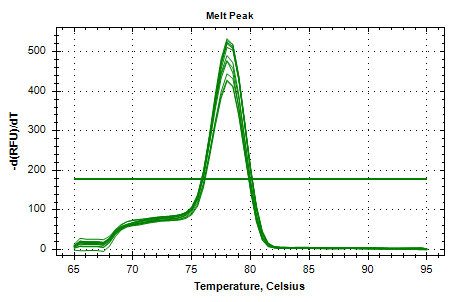** |
| *actb* | **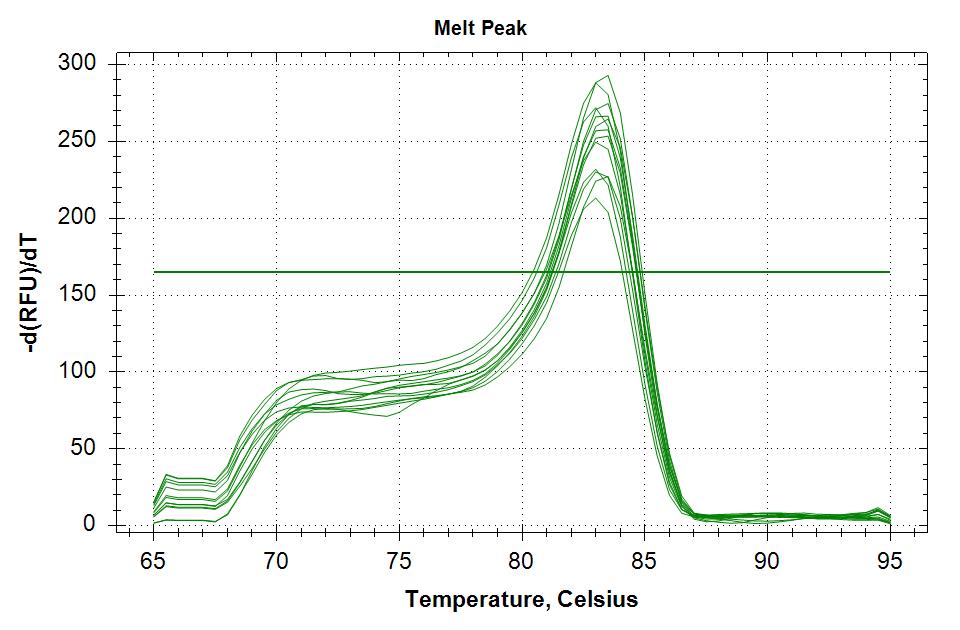** |
| *ef1a* | **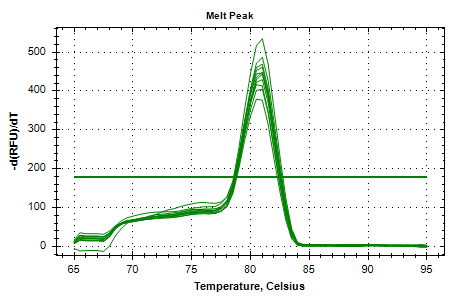** |
| *g6pd* | **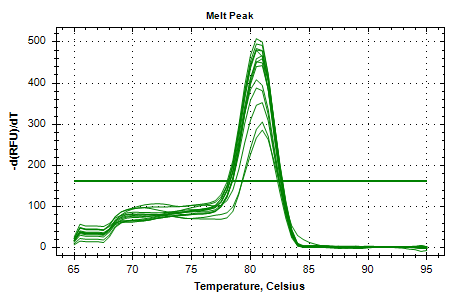** |
| *gapdh* | **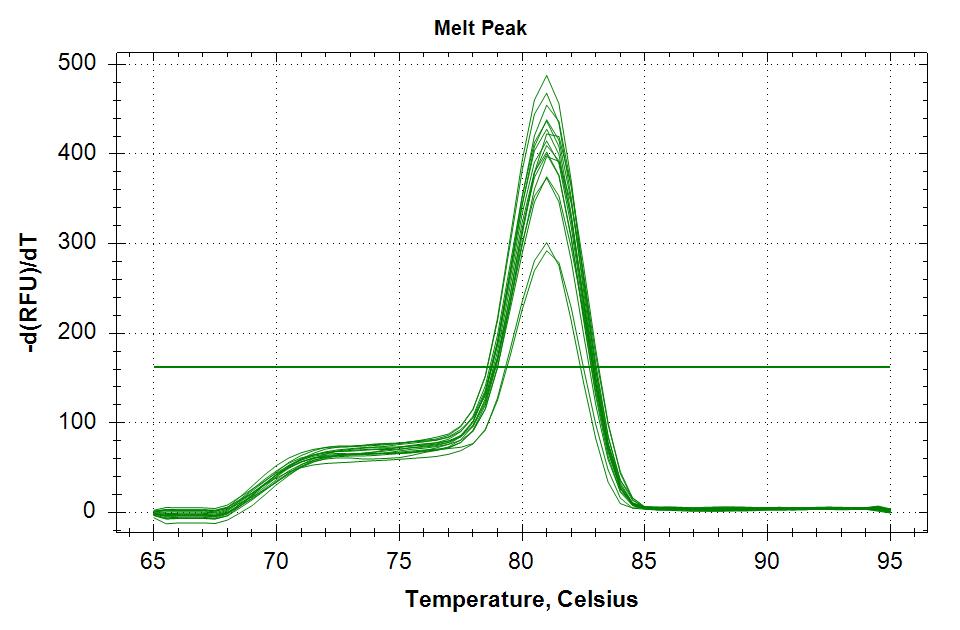** |
| *tbp* | **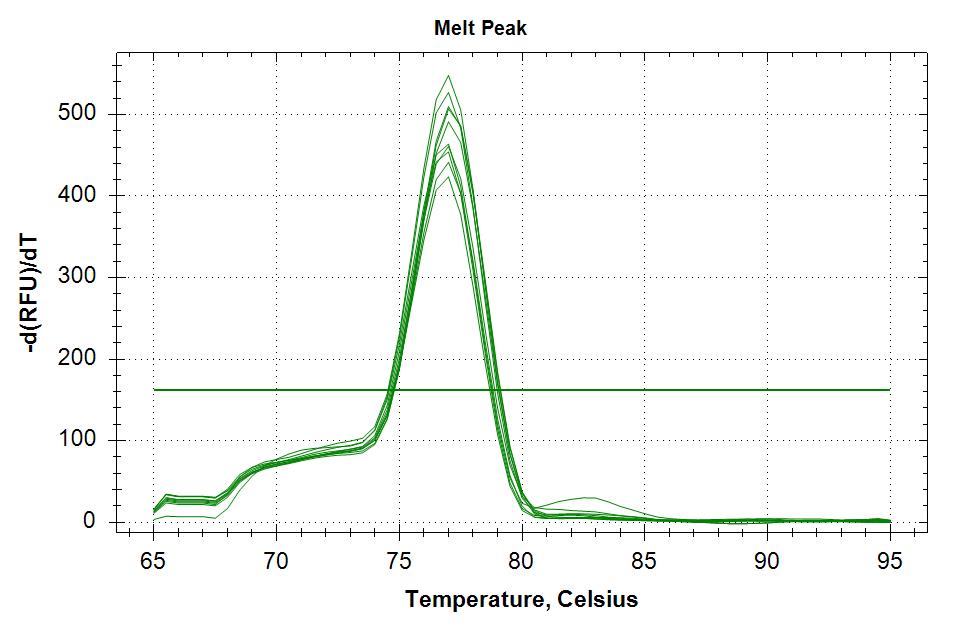** |
| *tubb2b* | **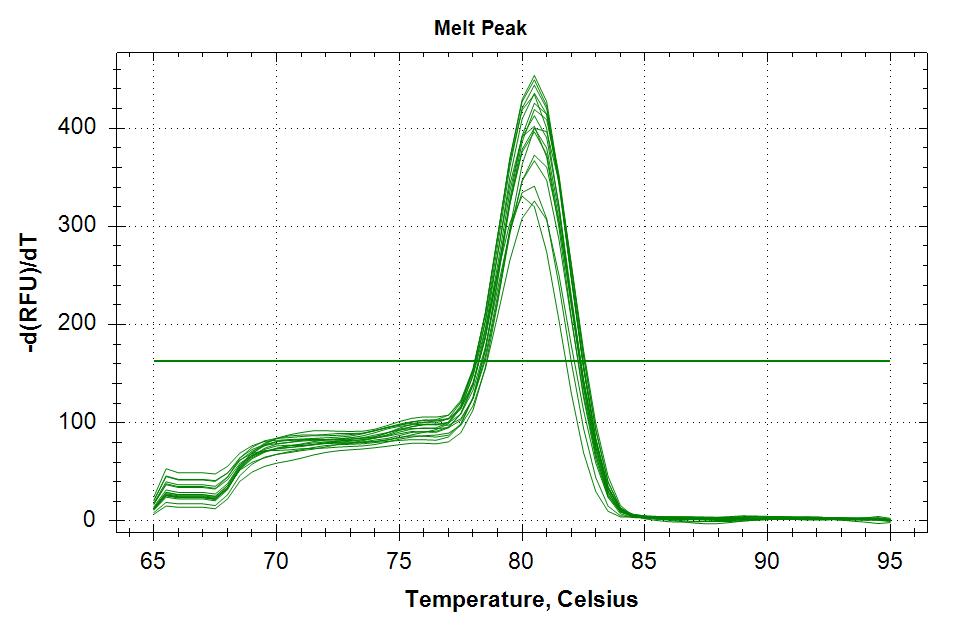** |
| *p53* | **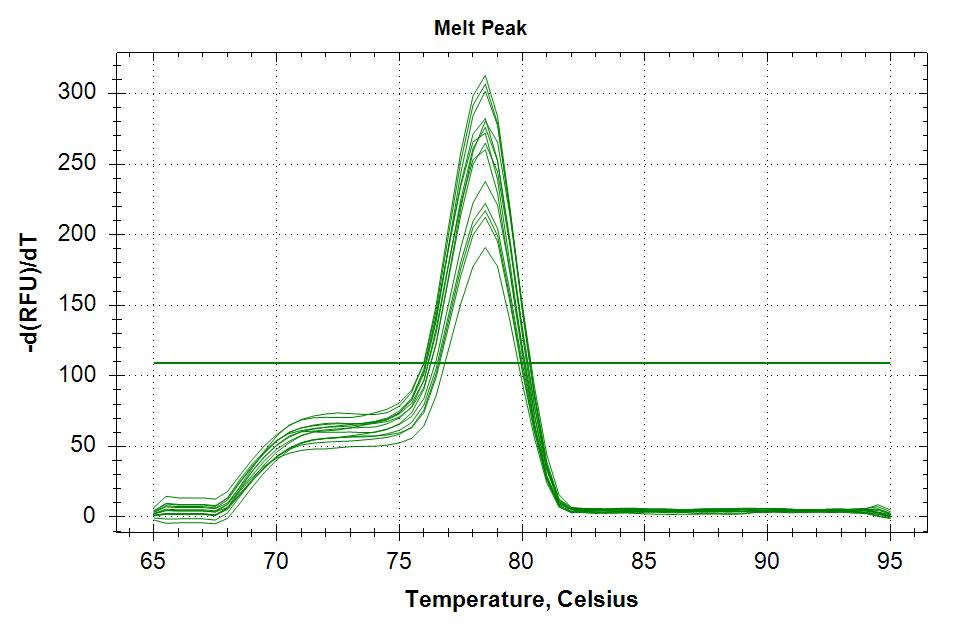** |
